# Protein Kinase C Iota Regulates Pancreatic Acinar-to-Ductal Metaplasia

**DOI:** 10.1371/journal.pone.0030509

**Published:** 2012-02-16

**Authors:** Michele L. Scotti, Kristin E. Smith, Amanda M. Butler, Shelly R. Calcagno, Howard C. Crawford, Michael Leitges, Alan P. Fields, Nicole R. Murray

**Affiliations:** 1 Department of Cancer Biology, Mayo Clinic, Jacksonville, Florida, United States of America; 2 Department of Pharmacological Sciences, Stony Brook University, Stony Brook, New York, United States of America; 3 Biotechnology Centre of Oslo, University of Oslo, Oslo, Norway; Technische Universität München, Germany

## Abstract

Pancreatic acinar-to-ductal metaplasia (ADM) is associated with an increased risk of pancreatic cancer and is considered a precursor of pancreatic ductal adenocarcinoma. Transgenic expression of transforming growth factor alpha (TGF-α) or K-ras^G12D^ in mouse pancreatic epithelium induces ADM *in vivo*. Protein kinase C iota (PKCι) is highly expressed in human pancreatic cancer and is required for the transformed growth and tumorigenesis of pancreatic cancer cells. In this study, PKCι expression was assessed in a mouse model of K-ras^G12D^-induced pancreatic ADM and pancreatic cancer. The ability of K-ras^G12D^ to induce pancreatic ADM in explant culture, and the requirement for PKCι, was investigated. PKCι is elevated in human and mouse pancreatic ADM and intraepithelial neoplastic lesions *in vivo*. We demonstrate that K-ras^G12D^ is sufficient to induce pancreatic ADM in explant culture, exhibiting many of the same morphologic and biochemical alterations observed in TGF-α-induced ADM, including a dependence on Notch activation. PKCι is highly expressed in both TGF-α- and K-ras^G12D^-induced pancreatic ADM and inhibition of PKCι significantly reduces TGF-α- and K-ras^G12D^-mediated ADM. Inhibition of PKCι suppresses K-ras^G12D^–induced MMP-7 expression and Notch activation, and exogenous MMP-7 restores K-ras^G12D^–mediated ADM in PKCι-depleted cells, implicating a K-ras^G12D^-PKCι-MMP-7 signaling axis that likely induces ADM through Notch activation. Our results indicate that PKCι is an early marker of pancreatic neoplasia and suggest that PKCι is a potential downstream target of K-ras^G12D^ in pancreatic ductal metaplasia *in vivo*.

## Introduction

Oncogenic *KRAS* mutations are found in >90% of pancreatic ductal adenocarcinomas (PDACs). [1] Mutational activation of *KRAS* is thought to occur early in PDAC development, as *KRAS* mutations are observed in ∼30% of PDAC precursor lesions, pancreatic intraepithelial neoplasia (PanIN). [1] A mouse model for conditional expression of an activated *Kras* (*Kras^G12D^*) allele in the pancreas from its physiological promoter has been utilized to investigate the role of oncogenic K-ras in initiation and progression of PDAC. [2], [3], [4] Expression of oncogenic K-ras induces formation of preneoplastic lesions in mice that are histologically similar to human PanINs (mouse PanINs, mPanINs). [2], [4] K-ras^G12D^–induced mPanINs become increasingly dysplastic, with a small percent progressing to invasive and metastatic adenocarcinomas, strongly suggesting that acquisition of an oncogenic *Kras* mutation can be an initiating event in pancreatic cancer. [2], [4]

Acinar-to-ductal metaplasia (ADM), the replacement of acinar cells with metaplastic ductal cells, is thought to be a source of neoplasia in the initiation of human PDAC. [4], [5], [6] Dysplastic features often arise in areas of ductal metaplasia, and metaplastic ductal cells exhibit many properties of embryonic progenitor cells, including Nestin expression. [4], [7], [8] The K-ras^G12D^-initiated mouse model of PDAC exhibits morphological, molecular and biochemical features indicative of ADM as early as 4 weeks of age, prior to the development of mPanINs. [2], [4] Aberrant activation of EGFR signaling in mouse pancreas also induces ADM and subsequent formation of PDAC. [7], [9], [10] EGFR-mediated ADM has been further characterized in an explant model. [11], [12] TGF-α induces primary mouse pancreatic acinar cells to transition through a de-differentiated, Nestin-positive intermediate to form metaplastic ductal structures. [7], [11], [12] Additional studies revealed that Notch signaling is both necessary and sufficient for acinar cell de-differentiation, Nestin expression and ADM in explant culture. [2], [12] MMP-7, which is also upregulated in human and mouse PanINs and PDAC, promotes activation of Notch signaling and ADM. [13], [14] MMP-7 is required for ADM in explant culture, and expression of a constitutively active Notch construct reconstitutes ADM in MMP-7–depleted acinar cells, indicating that MMP-7-dependent Notch activity is required for ADM. [14] These studies demonstrate the utility of the pancreatic acinar cell explant model for characterization of ADM, and strengthen the link between pancreatic metaplasia, neoplasia and initiation of PDAC.

We have identified PKCι as an important effector in oncogenic K-ras-induced transformation of lung and intestinal epithelial cells. [15], [16] We have also demonstrated that PKCι expression is elevated in a large percent of primary pancreatic adenocarcinomas, and high PKCι expression predicts poor patient survival. [17] In the current study, we demonstrate that PKCι is elevated in pancreatic metaplasia associated with human PDAC tumors and in K-ras^G12D^-mediated pancreatic metaplasia *in vivo*. To further characterize the molecular mechanism of K-ras^G12D^-mediated pancreatic ADM we employed a well-characterized mouse pancreatic acinar cell explant model. In this context, we evaluated the role of PKCι in K-ras^G12D^-mediated pancreatic ADM. Expression of oncogenic K-ras, the most frequently mutated oncogene in PDAC, is sufficient to induce pancreatic ADM in explant culture. PKCι expression is elevated in K-ras^G12D^- and TGFα-induced ADM. Inhibition of PKCι significantly reduces both K-ras^G12D^- and TGFα-induced ADM and also significantly reduces K-ras^G12D^-mediated Nestin expression, Notch activation and MMP-7 expression. Exogenous MMP-7 partially but significantly reconstitutes K-ras^G12D^-mediated ADM in PKCι-depleted cells, suggesting that PKCι mediates initiation of ADM, at least in part, by regulating MMP-7 expression. Our results demonstrate that K-ras^G12D^-mediated ADM in explant culture is regulated by PKCι.

## Results

### PKCι is induced in oncogenic K-ras-mediated ADM *in vivo*

**Figure 1:**
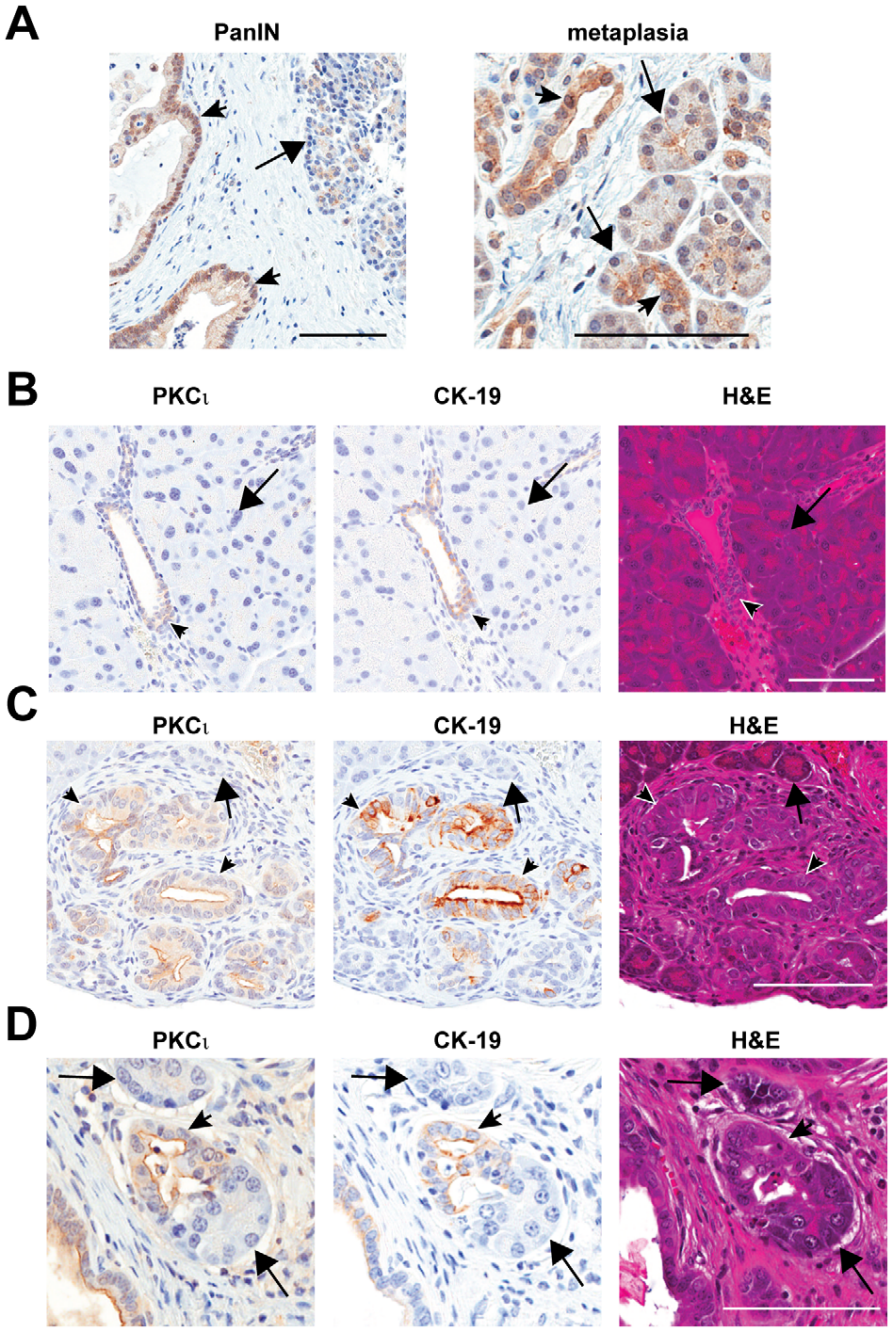
PKCι expression is elevated in PanINs and pancreatic metaplastic ducts. **A**) Immunohistochemical detection of PKCι (brown) in formalin-fixed human pancreatic tumor-associated PanIN (left) and metaplastic ducts (right). Arrowhead = PanIN (left), metaplastic duct (right); Arrow = cell with acinar morphology. **B**) Immunohistochemical detection of PKCι (brown) and CK19 (brown) in serial sections of WT mouse pancreas. H&E staining demonstrates tissue morphology. Arrowhead = pancreatic duct, Arrow = normal acinar cells. **C, D**) Immunohistochemical detection of PKCι and CK19 expression in serial sections of pancreatic epithelium of a *P48-Cre*;*LSL*-*Kras* mouse. [2] H&E staining demonstrates tissue morphology. Arrows = cells with acinar morphology; Arrowheads = K-ras^G12D^-induced *C)* mPanINs or *D)* metaplastic ducts. Scale bars, 100 µm.

PKCι expression is elevated in the vast majority of primary PDAC, and high PKCι expression predicts poor patient survival. [17] PKCι is also elevated in PanINs and pancreatic metaplastic ducts associated with human PDAC (**Figure 1A**). In normal mouse pancreas, PKCι is detected in interlobular ductal cells, but not in acinar cells (**Figure 1B**). PKCι expression was also detected in mPanINs (**Figure 1C**) from *P48-Cre*;*LSL*-*Kras* mice. PKCι expression tended to increase, with a redistribution from apical to cytoplasmic localization, in more progressed mPanIN lesions and in adenocarcinoma (**Figure S1**). Interestingly, PKCι was also expressed in the metaplastic ductal cells, but not in the morphologically normal acinar cells of K-ras^G12D^-induced ADM (**Figure 1D**). K-ras^G12D^-induced pancreatic ADM exhibits some of the same properties of mPanINs, including increased proliferation and Notch signaling, [2], [4], [11], [12], [14] suggesting ADM is a precursor to mPanINs and therefore relevant to the initiation of PDAC. [4] The increased PKCι expression observed in K-ras^G12D^-induced ADM prompted us to investigate a possible role for PKCι in K-ras^G12D^-induced ADM using an explant culture amenable to evaluation of the molecular mechanisms involved in the specific transdifferentiation of pancreatic acinar cells to metaplastic duct-like cells.

### PKCι regulates TGF-α-mediated ADM

**Figure 2:**
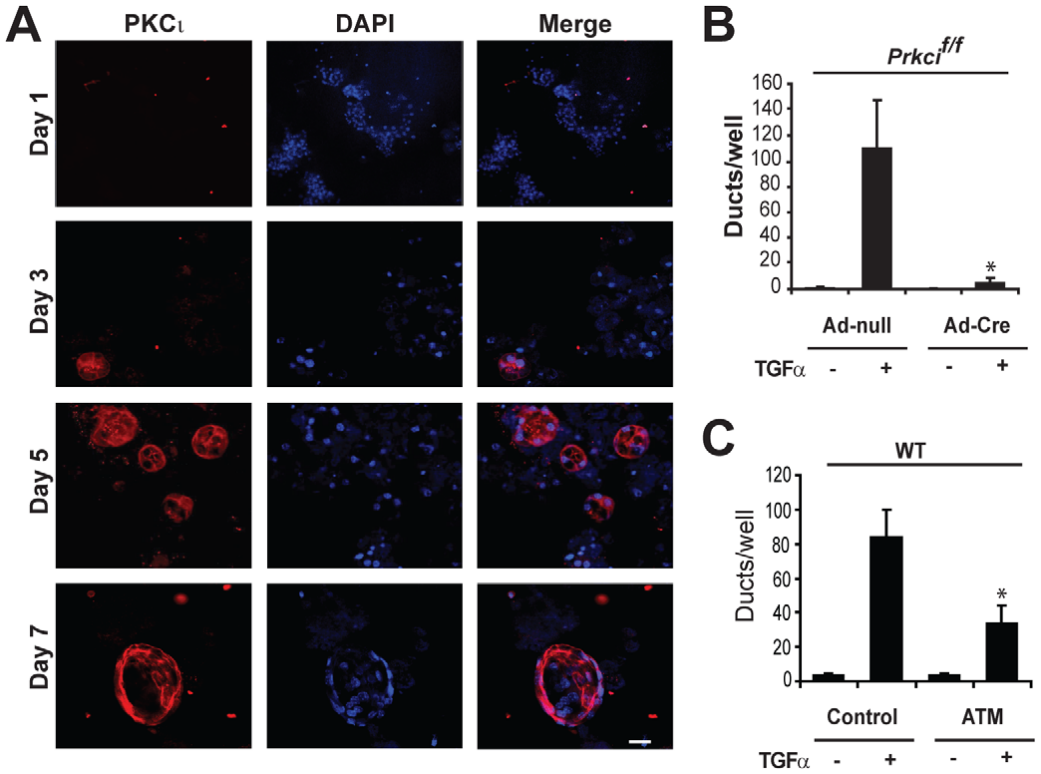
PKCι regulates TGF-α-induced formation of metaplastic ducts. WT pancreatic acinar cells were embedded in collagen matrix with 50 ng/ml TGF-α. **A**) PKCι immunofluorescence (red) in explant cultures on days 1, 3, 5 and 7. PKCι was detected in ductal cells but not acinar cells. Cells were co-stained with DAPI (blue) to define cell nuclei. Scale bar, 50 µm. **B–C**) Quantitative analysis of TGF-α-induced duct formation in pancreatic explants in which PKCι is genetically or pharmacologically inhibited. **B**) Pancreatic acinar cells were isolated from *Prkci^f/f^* mice, incubated with control, adeno-null virus (Ad-null) or adeno-Cre virus (Ad-Cre) and embedded in collagen ± TGF-α for 7 days. **C**) Pancreatic acinar cells isolated from WT mice were embedded in collagen ± TGFα and ±100 µM aurothiomalate (ATM) for 7 days. Plots are an average of three independent experiments. Bars = mean ± SEM and *P<.05 versus TGF-α-treated control wells.

As described, mouse pancreatic acinar cells plated in collagen matrix undergo TGF-α-induced ADM, characterized by morphological conversion from clusters of zymogen-containing acinar cells to cystic structures with a ductal morphology (**Figure S2A**). [11], [14] This morphological transformation is associated with a loss of acinar differentiation, as assessed by amylase expression and a concomitant increase in ductal differentiation, characterized by expression of cytokeratin 19 (CK-19) (**Figure S2B**). [11] PKCι expression is undetectable in isolated acinar cells, but is significantly increased as cells undergo TGF-α-induced ADM (**Figure 2A**), consistent with PKCι playing a role in the transdifferentiation of pancreatic acinar cells to metaplastic ducts.

To investigate the role of PKCι in TGF-α-mediated ADM, we utilized pancreatic acinar cells isolated from *Prkci^f/f^* mice. [18] *Prkci^f/f^* acinar cells were transduced with control adeno-virus (adeno-null) or adeno-virus expressing Cre-recombinase (adeno-Cre) to induce genetic recombination and deletion of the loxP-flanked *Prkci* allele (**Figure S3A**). Adeno-null-treated *Prkci^f/f^* acinar cells underwent ADM in response to TGF-α, while adeno-Cre-treated *Prkci^f/f^* acinar cells were largely refractory to TGF-α-induced ADM (**Figure 2B**). Adeno-Cre treatment did not inhibit TGF-α-mediated ADM in *R26R* acinar cells (**Figure S3B and C**). Consistent with a specific requirement for PKCι, addition of the molecularly-targeted inhibitor of PKCι signaling, aurothiomalate, [19], [20], [21] to the explant culture significantly reduced TGF-α-induced ADM (**Figure 2C**). These data demonstrate at least a partial requirement for PKCι for TGF-α-induced ADM.

### K-ras^G12D^ induces ADM in explant culture

**Figure 3:**
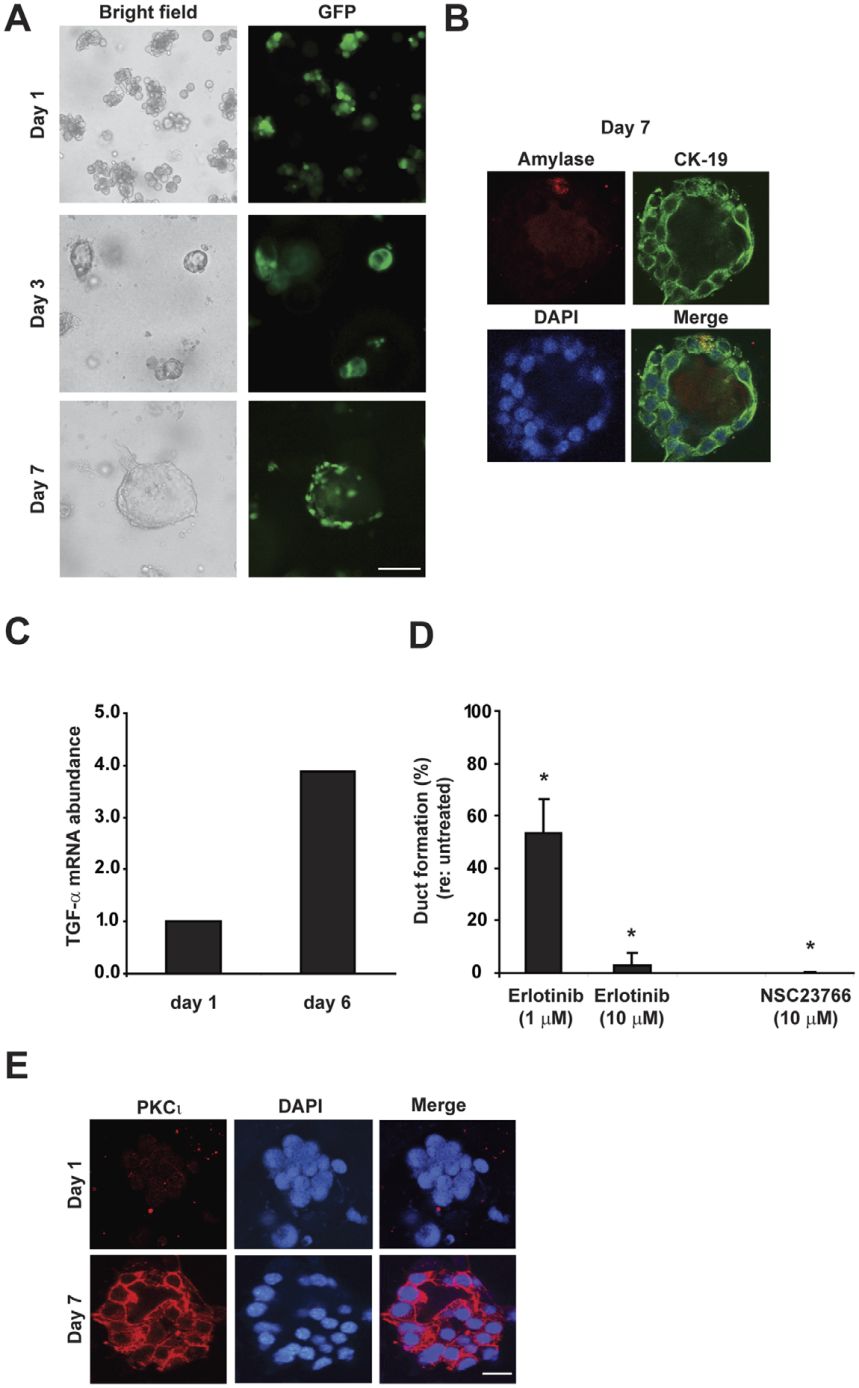
K-ras^G12D^ induces ADM in explant culture. Pancreatic acinar cells were isolated from *LSL-Kras* mice, incubated with adeno-Cre-GFP virus, and embedded in collagen (without exogenous TGF-α). **A**) Representative bright field and fluorescent images were captured on days 1, 3 and 7. GFP fluorescence indicates infection by adeno-Cre-GFP virus. Scale bar, 200 µm. **B**) ADM was confirmed by co-immunofluorescence of amylase (red) and CK-19 (green) in K-ras^G12D^–induced ductal cells on day 7. **C**) mRNA was isolated from day 1 and 6 explant cultures of Ad-Cre virus-treated *LSL-Kras* acinar cells and analyzed by qPCR for TGF-α expression. Data is presented relative to 18S abundance (×10^5^) and is representative of two independent experiments. **D**) Pancreatic acinar cells were isolated from *LSL-Kras* mice, incubated with Ad-Cre and embedded in collagen ± 1 µM or 10 µM Erlotinib, or 10 µM NSC23766 for 5 days. Quantitative analysis of metaplastic duct formation is plotted for each treatment. Bars = mean ± SD. *P<0.05 (Student *T*-test). Plots are representative of two independent experiments. **E**) PKCι (red) was undetectable in *LSL-Kras* explant culture on day 1, but was elevated in K-ras^G12D^–induced ductal cells on day 7. Scale bar, 25 µm.

The earliest morphological alteration observed in the pancreata of *P48-Cre*;*LSL*-*Kras* mice is the formation of metaplastic structures containing both acinar- and duct-like cells. [4] Molecular analysis of these metaplastic structures suggests that K-ras^G12D^ induces ADM. [4] To evaluate the role of PKCι in K-ras^G12D^-induced ADM, we first characterized the ability of K-ras^G12D^ to induce ADM in explant culture. Pancreatic acinar cells were isolated from *LSL*-*Kras* mice and incubated with adeno-Cre-GFP to induce genomic recombination (**Figure S4A**) and expression of K-ras^G12D^. K-ras^G12D^ was sufficient to induce ADM in explant culture in the absence of exogenous TGF-α, as determined by transition from acinar to ductal morphology (**Figure 3A**) with a single layer of cells surrounding a clear lumen, indicative of a mature ductal structure (**Figure S4B**). Likewise, a loss of expression of acinar cell markers and a gain of expression of ductal cell markers was also observed in K-ras^G12D^–induced ADM (**Figure 3B** and **Figure S4C**) confirming transition from acinar to ductal gene expression profile.

While K-ras^G12D^ induced ADM in explant culture in the absence of exogenous TGF-α, TGF-α mRNA was elevated in K-ras^G12D^–mediated ADM (**Figure 3C**). K-ras^G12D^–induced ADM was partially, but significantly reduced by Erlotinib, an EGFR inhibitor (**Figure 3D**). Furthermore, inhibition of Rac1 blocks K-ras^G12D^-mediated ADM (**Figure 3D**), consistent with a recent report that Rac1 activity regulates ADM. [22] K-ras^G12D^-induced ADM was also accompanied by a significant increase in PKCι expression (**Figure 3E**) in CK-19-positive duct cells (**Figure S4D**). Taken together, these results demonstrate that K-ras^G12D^ induces metaplastic duct formation in explant culture, as in mouse pancreas *in vivo*, [4] and that PKCι expression is induced in K-ras^G12D^-mediated metaplastic ducts *in vitro* and *in vivo*.

### PKCι regulates K-ras^G12D^-induced ADM

**Figure 4:**
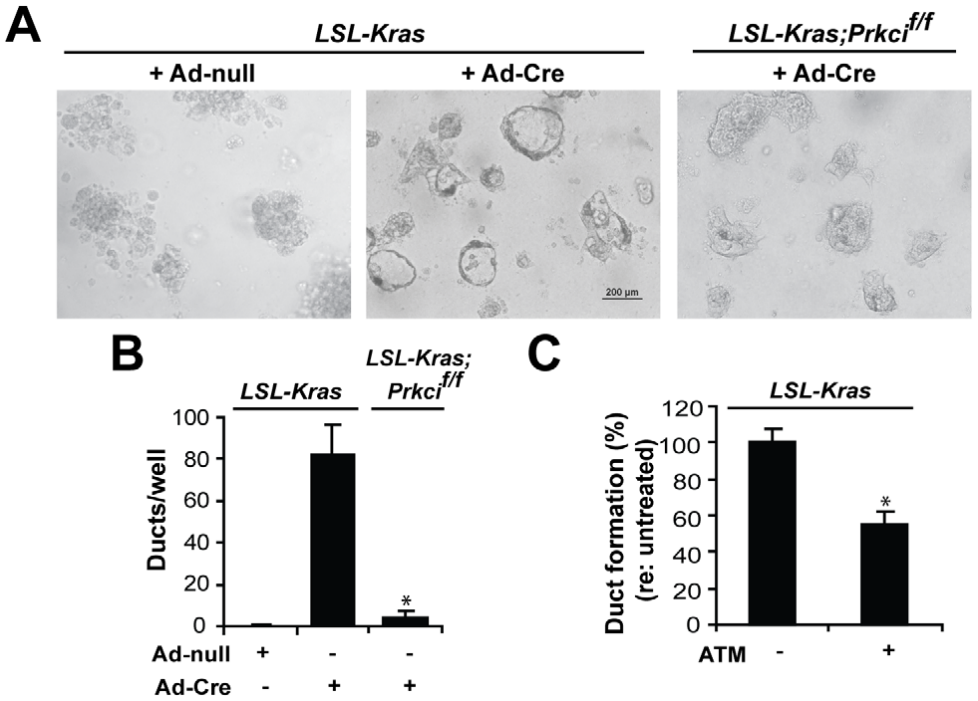
PKCι regulates K-ras^G12D^-induced ADM. **A, B**) Pancreatic acinar cells from *LSL-Kras* and *LSL-Kras;Prkci^f/f^* mice were incubated with Ad-null or Ad-Cre and embedded in collagen. Cultures were **A**) photographed on day 7 (Scale bar, 200 µm) and **B**) quantified for metaplastic duct formation. **C**) Pancreatic acinar cells from *P48*-Cre;*LSL-Kras* mice were embedded in collagen ± 100 µM aurothiomalate (ATM). **B, C**) Quantitative analysis of metaplastic duct formation is plotted. Plots are the average of three independent experiments. Bars = mean ± SEM and *P<.05 versus *LSL-Kras*.

We next tested the hypothesis that PKCι plays a role in K-ras^G12D^-induced ADM in explant culture, using acinar cells from *LSL-Kras;Prkci^f/f^* mice which allow simultaneous Cre-mediated activation of expression of K-ras^G12D^ and genetic knockout of PKCι. [16] K-ras^G12D^ induced ADM in *LSL*-*Kras* acinar cells, but not *LSL-Kras;Prkci^f/f^* acinar cells (**Figure 4A, B**). Expression of PKCι and CK-19 remained low in adeno-Cre-GFP-treated *LSL-Kras;Prkci^f/f^* explant cultures, compared to adeno-Cre-GFP-treated *LSL-Kras* explant cultures (compare **Figure S5A** to **Figure S4D**). GFP expression confirmed highly efficient viral infection of both *LSL-Kras* and *LSL-Kras;Prkci^f/f^* acinar cells (**Figure S5B**) and PCR analysis demonstrated adeno-Cre-mediated recombination of both the *LSL-Kras* and *Prkci^f/f^* floxed alleles in the *LSL-Kras;Prkci^f/f^* acinar cells (**Figure S5C**). Furthermore, addition of aurothiomalate to the explant culture also significantly reduced K-ras^G12D^-mediated ADM (**Figure 4C**), without a significant effect on cell viability (data not shown). aurothiomalate did not prevent K-ras^G12D^-induced PKCι expression (**Figure S5D and E**), however, PKCι was detected primarily in the cytoplasm of aurothiomalate-blocked acinar-like cells, in contrast to the more basolateral localization of PKCι in K-ras^G12D^-induced metaplastic ducts (**Figure S5D**). Therefore, genetic and pharmacological inhibition of PKCι significantly reduce K-ras^G12D^-mediated ADM, strongly supporting a role for PKCι activity in K-ras^G12D^-mediated ADM.

### PKCι promotes Notch activation and formation of a Nestin-positive intermediate in K-ras^G12D^-expressing acinar cells

**Figure 5:**
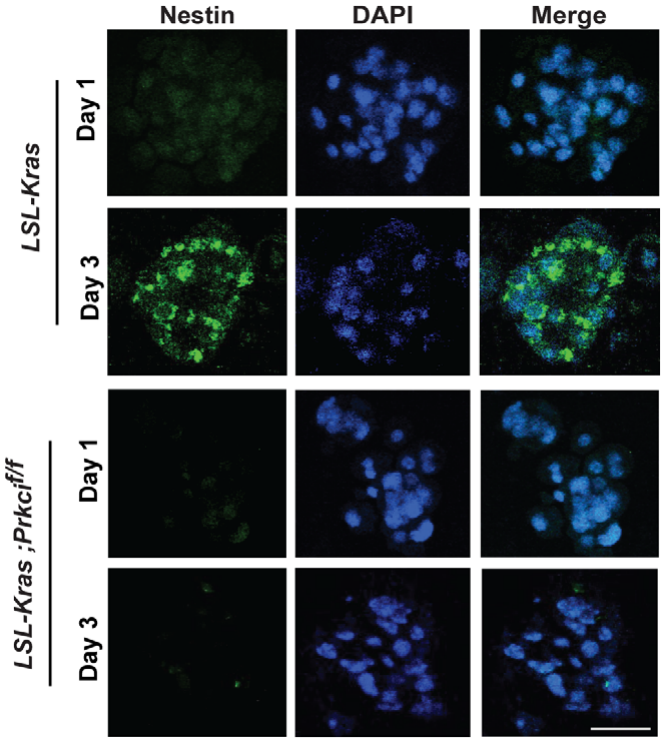
Inhibition of PKCι significantly reduces K-ras^G12D^-induced formation of Nestin-positive intermediate. Pancreatic acinar cells isolated from *LSL-Kras* and *LSL-Kras;Prkci^f/f^* mice were incubated with Ad-Cre and embedded in collagen. Nestin immunofluorescence (green) was very low on day 1, and induced on day 3 of explant culture in *LSL-Kras* but not *LSL-Kras;Prkci^f/f^* cells. Cells were co-stained with DAPI (blue). Scale bar, 25 µm.

TGF-α-induced ADM proceeds through a de-differentiated, Nestin-positive intermediate that requires activation of Notch. [11], [12], [14] We asked whether K-ras^G12D^-induced ADM also proceeds through a Nestin-positive intermediate. Nestin expression was undetectable in K-ras^G12D^-expressing explant cultures on day 1, but increased significantly by day 3 (**Figure 5**), similar to the kinetics of Nestin expression in TGFα-induced ADM. [11], [12], [14] PKCι ablation blocked K-ras^G12D^-induced Nestin expression on day 3 (**Figure 5**), implicating PKCι in the initial de-differentiation step of K-ras^G12D^-induced ADM.

**Figure 6:**
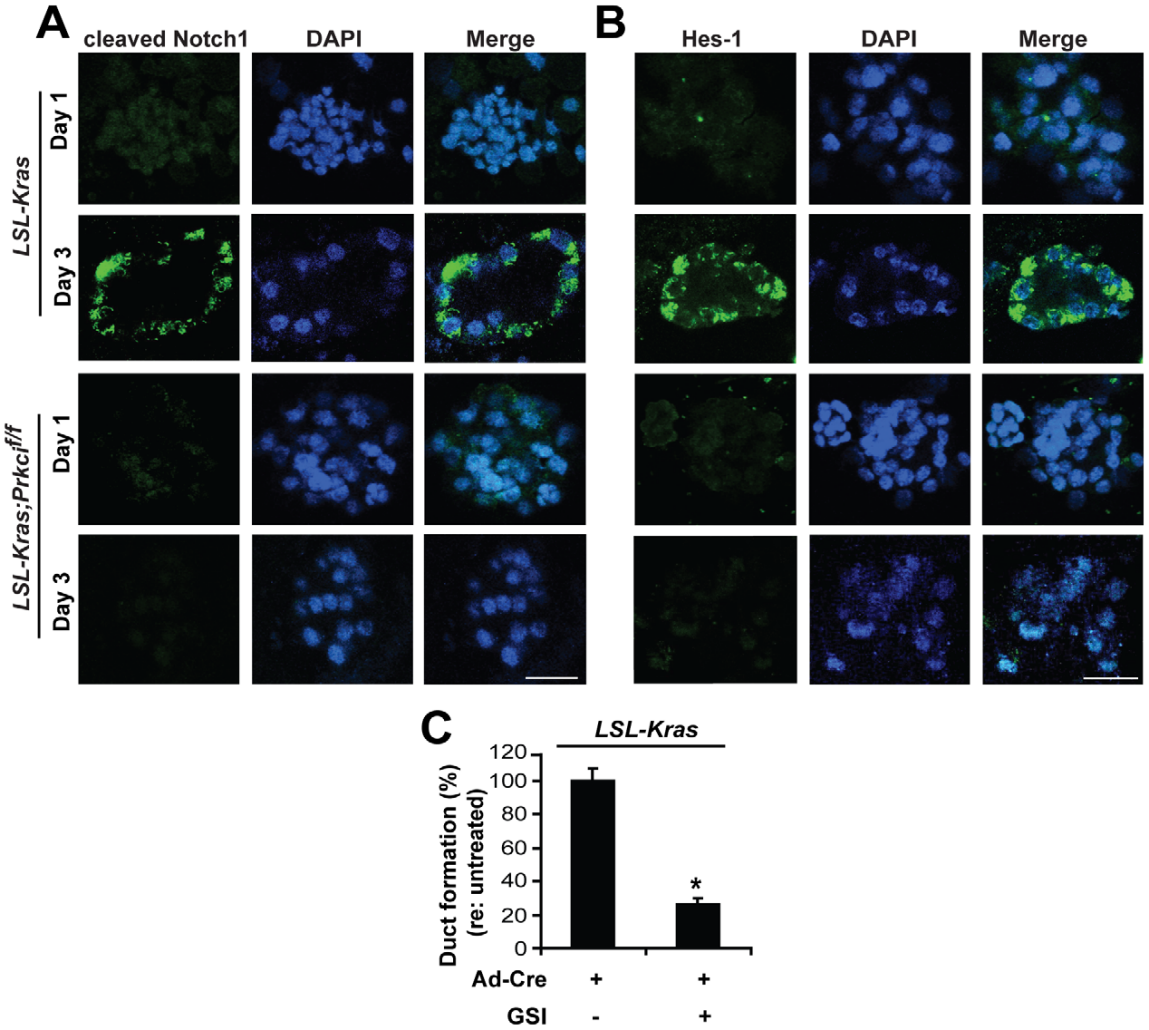
Inhibition of PKCι significantly reduces activation of Notch signaling. **A, B**) Pancreatic acinar cells isolated from *LSL-Kras* and *LSL-Kras;Prkci^f/f^* mice were incubated with Ad-Cre and embedded in collagen matrix. Pancreatic explants were stained for **A**) cleaved Notch1 (green) and **B**) Hes1 (green). Cultures were co-stained with DAPI (blue). Scale bar, 25 µm. **C**) A γ-secretase inhibitor significantly reduced K-ras^G12D^-induced metaplastic duct formation. Pancreatic acinar cells isolated from *LSL-Kras* mice were incubated with Ad-Cre and embedded in collagen ± 1 µM L-685,458. Quantitative analysis of duct formation is plotted. Plot is representative of two independent experiments. Bars = mean ± SEM and *P<.05.

Notch signaling is activated in K-ras^G12D^-mediated ADM *in vivo*, [4] and is both required and sufficient to induce pancreatic ADM in explant culture. [12] We therefore evaluated whether Notch was activated by K-ras^G12D^ in explant culture (**Figure 6**). Gamma-secretase-dependent cleavage of the Notch receptor is required for activation of Notch signaling. [23] Using an antibody specific for gamma-secretase cleaved (activated) Notch1, we detected little to no activated Notch1 in K-ras^G12D^-expressing acinar cell explant culture on day 1, but by day 3 the amount of activated Notch was significantly increased (**Figure 6A**). K-ras^G12D^-induced Notch1 activation was inhibited in PKCι–deficient cells (**Figure 6A**). Likewise, expression of Hes1, a Notch transcriptional target, was induced in K-ras^G12D^-expressing explant culture, but the increased Hes1 expression was blocked by loss of PKCι expression (**Figure 6B**), implicating PKCι in the regulation of Notch1 activation. Finally, K-ras^G12D^-induced ADM was significantly reduced by a gamma-secretase inhibitor (L-685,458)[24] (**Figure 6C**), suggesting that K-ras^G12D^-induced ADM may require Notch activity.

### MMP-7 overcomes PKCι deficiency to recover ADM

**Figure 7:**
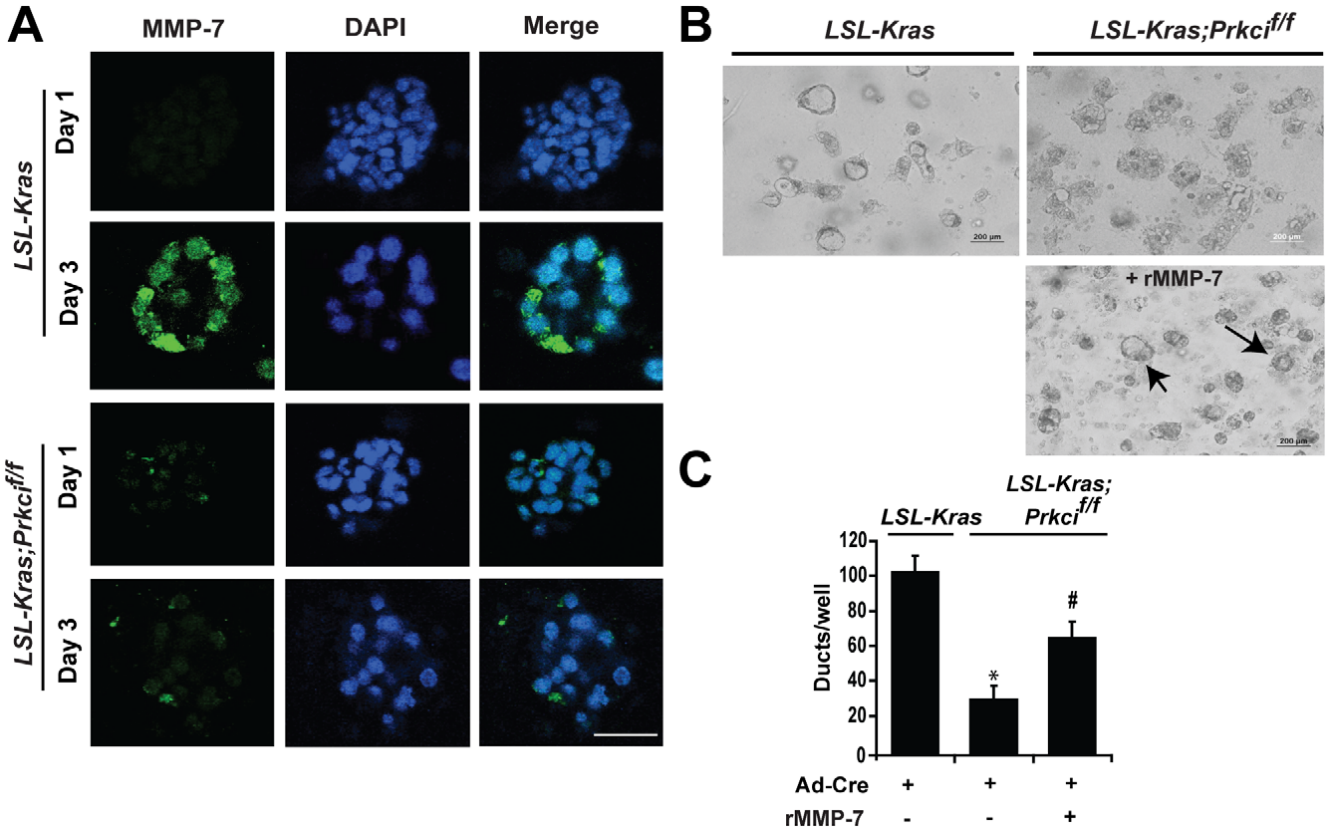
MMP-7 is induced by K-ras^G12D^ and exogenous MMP-7 partially rescues ADM in PKCι-depleted pancreatic acinar cells. Pancreatic acinar cells were isolated from *LSL-Kras* and *LSL-Kras;Prkci^f/f^* mice, incubated with Ad-Cre and embedded in collagen. **A**) Pancreatic explants were stained for MMP-7 (green) on day 1 and 3. Scale bar, 25 µm. **B**) Representative bright field images of Ad-Cre-infected cultures of *LSL-Kras* cells, *LSL-Kras;Prkci^f/f^* cells and *LSL-Kras;Prkci^f/f^* cells incubated with 200 ng/ml active recombinant MMP-7 (rMMP-7). Arrows indicate restored metaplastic duct formation in MMP-7-treated *LSL-Kras;Prkci^f/f^* cultures. Scale bar, 200 µm. **C**) Quantitative analysis of duct formation in *B)*. Bars = mean ± SEM. *P<.05 versus *LSL-Kras*; #*P*<.05 versus *LSL-Kras* and *LSL-Kras;Prkci^f/f^* without rMMP-7. Plot is representative of two independent experiments.

Our data strongly suggest that PKCι regulates acinar-to-ductal transdifferentiation prior to Notch activation. Sawey et al. demonstrated that MMP-7 is both necessary and sufficient for Notch activation in ADM in explant culture. [14] MMP-7 expression is elevated in K-ras^G12D^-induced mPanINs *in vivo*, suggesting a role for MMP-7 in K-ras^G12D^-initiated neoplasia. [2] Consistent with these findings, we found that K-ras^G12D^-induced ADM was accompanied by a significant increase in MMP-7 expression, whereas PKCι-null explants showed no induction of MMP-7 (**Figure 7A**). Genetic knockout of PKCι expression in K-ras^G12D^-expressing explant culture significantly reduced the K-ras^G12D^-induced increase in MMP-7 mRNA expression (**Figure S6A**), suggesting that PKCι may regulate MMP-7 transcription. To test whether restoration of MMP-7 rescues K-ras^G12D^-induced ADM in PKCι-deficient acinar cells, we added recombinant MMP-7 to the explant culture. Indeed, MMP-7 significantly enhanced ADM in PKCι-deficient cells (**Figure 7B, C**). PKCι expression remains low in MMP-7-induced ducts (compare **Figure S6B** to **Figure S5A**), suggesting that addition of exogenous MMP-7 by-passes PKCι in promoting ADM, and providing support for the hypothesis that PKCι regulates ADM, at least in part, by controlling MMP-7 expression. [14] The lack of complete reconstitution of ADM by MMP-7 in PKCι deficient acinar cells may be due to the reduced diffusion of MMP-7 in collagen matrix, but may also indicate the requirement of additional factors downstream of PKCι. Our results demonstrate that K-ras^G12D^-induced ADM utilizes signaling pathways implicated in TGF-α-induced ADM in explant culture and K-ras^G12D^-induced pancreatic carcinogenesis *in vivo*. [2], [4], [25] Importantly, we make the novel observation that PKCι regulates K-ras^G12D^- and TGF-α-mediated pancreatic ADM in explant culture.

## Discussion

PKCι is highly overexpressed in human pancreatic cancer and expression of PKCι-targeted RNAi significantly reduces PDAC cell transformed growth and tumorigenicity *in vivo*. [17] These data suggest that PKCι plays a required role in human pancreatic cancer. We have previously defined a required role for PKCι in oncogenic K-ras-mediated initiation of preneoplastic lesions of the lung and intestinal epithelium. [15], [16] In this study, we investigated the role of PKCι in oncogenic K-ras signaling and initiation of pancreatic metaplasia using a well-characterized pancreatic explant culture model.

Increasing evidence suggest that PanINs can develop from acinar cells and that ADM may be a critical intermediate in the development of PanINs. [4], [26] PKCι expression is significantly higher in K-ras^G12D^-mediated ductal metaplasia than in morphologically normal regions of mouse pancreatic acinar cells, and remains elevated in mPanINs and adenocarcinoma. To directly investigate the role of PKCι in K-ras^G12D^-mediated ADM, we utilized an acinar cell explant model of ADM [11] in which TGF-α induces acinar cell de-differentiation to Nestin-positive, precursor-like intermediates that subsequently convert to cytokeratin-expressing metaplastic ducts. [12], [14] Indeed, several studies have concluded that the rate limiting step in K-ras^G12D^-mediated mPanIN formation appears to be de-differentiation of mature pancreatic exocrine cells. For example, creating an expanded, de-differentiated cell population through genetic knockout of Mist1 (an acinar cell-restricted transcription factor) or pancreatic injury, enhanced the rate of formation of K-ras^G12D^-mediated mPanINs. [27], [28] Likewise, targeting K-ras^G12D^ only to Nestin-expressing progenitor cells yielded similar levels of mPanINs as targeting the entire exocrine cell population, [3] suggesting that this de-differentiated, progenitor-like population of cells may be the target for K-ras^G12D^-mediated initiation of PDAC.

In this study, we demonstrate that K-ras^G12D^ induces ADM in explant culture in a manner similar to TGF-α-induced ADM, including progression through a Nestin-positive intermediate and a dependence on PKCι. Inhibition of PKCι significantly reduced K-ras^G12D^-induced Nestin expression, suggesting a role for PKCι in K-ras^G12D^-mediated de-differentiation of mature acinar cells. K-ras^G12D^-induced ADM does not require exogenous TGF-α, however, activation of K-ras^G12D^ induced TGF-α mRNA expression and inhibition of EGFR decreased K-ras^G12D^–induced ADM in explant culture. Since EGFR expression and activation is induced in K-ras^G12D^–induced ADM *in vivo* [4] our data suggests that K-ras^G12D^ may induce ADM, at least in part by up-regulation of autocrine EGFR signaling. This hypothesis is supported by the observation that EGFR signaling synergizes with K-ras^G12D^ to promote progression of mPanINs in the *LSL-Kras* mouse model of pancreatic cancer. [29]

The Notch signaling pathway, which blocks pancreatic acinar cell differentiation and maintains cells in a non-differentiated, proliferative state, is required for normal pancreatic development. [30] Notch signaling is aberrantly reactivated in PanINs and PDAC, as well as K-ras^G12D^-initiated mPanINs. [12], [31] These observations suggest a required role for Notch signaling in K-ras^G12D^-mediated initiation of PDAC. Notch signaling is activated by TGF-α in mouse pancreas *in vivo* and in explant culture, and Notch signaling is required and sufficient for TGF-α-induced ADM in explant culture. [12], [14] K-ras^G12D^ also induces Notch activation in acinar cell explant culture, and K-ras^G12D^-mediated ADM is significantly reduced by a gamma-secretase inhibitor, suggesting that K-ras^G12D^-mediated ADM may require Notch activation. Inhibition of gamma-secretase activity, which blocks activation of Notch signaling, inhibits progression of K-ras-mediated mPanINs *in vivo* and reduces the transformed growth of pancreatic cancer cells. [25], [32] Likewise, expression of a constitutively-active Notch promoted formation and progression of K-ras-mediated mPanINs, suggesting a tumor-promotive role for Notch signaling in the mouse model of PDAC. [26] Conversely, genetic knockout of Notch1 expression promoted formation and progression of K-ras-mediated mPanINs, suggesting that under some conditions, or at certain stages of cancer development, Notch signaling may suppress pancreatic cancer. [33] In this context, it will be interesting to determine whether PKCι regulates Notch activation in mPanINs and PDAC, since PKCι remains elevated as mPanINs become increasingly dysplastic.

Inhibition of PKCι significantly reduced K-ras^G12D^-mediated MMP-7 expression, Notch activation and ADM in explant culture. Addition of exogenous MMP-7 to the explant culture partially, but significantly, recovered the inhibitory effect of PKCι deficiency. These results implicate MMP-7 as a likely downstream effector of PKCι in K-ras^G12D^-mediated ADM, and a possible mechanism by which PKCι regulates Notch1 activation, since MMP-7 can cleave and activate Notch1 in metaplastic acinar cells. [14]

PKCι is required for mutant *Apc*-induced intestinal adenoma formation. [34] Tumorigenesis in the *Apc^min/+^* mouse model also requires MMP-7 and Notch activation. [35], [36] MMP-7 has been identified as a target gene of Rac1 in colorectal carcinoma cells, [37] suggesting regulation of Rac1 activity as a possible mechanism by which PKCι may regulate MMP-7 expression and initiation of pancreatic and colon cancer. In addition, PKCι regulates expression of another MMP, MMP-10, in lung cancer cells. [38] Both PKCι and MMP-10 are required for lung cancer cell transformed growth, [38], [39] suggesting that regulation of expression of MMPs may be a general mechanism by which PKCι controls initiation and maintenance of the transformed phenotype in cancer.

In this study, we use both genetic and pharmacological means to demonstrate that PKCι regulates TGF-α- and K-ras^G12D^-induced ADM in explant culture. Our results indicate that PKCι is an early marker of pancreatic neoplasia. Our results further suggest that K-ras^G12D^-mediated ADM utilizes a PKCι-MMP-7 signaling pathway, and that, similar to lung and colon cancer, [15], [16] PKCι may play a promotive role in the initiation of PDAC. Tri-transgenic *P48-Cre;LSL-Kras;Prkci^f/f^* mice would be useful to test the hypothesis that PKCι is required for K-ras^G12D^-mediated ADM and mPanIN formation *in vivo*. However, these tri-transgenic mice are currently unavailable due to difficulties in breeding. Overcoming these breeding difficulties, whose cause is currently unknown, will be important for future studies to test the prediction of our in vitro results, namely, that PKCι plays a role in K-ras^G12D^-mediated pancreatic metaplasia and carcinogenesis in vivo.

## Materials and Methods

### Ethics Statement

Biospecimens were obtained from the Mayo Clinic SPORE in Pancreatic Cancer Tissue Core under an approved Mayo Clinic Institutional Review Board protocol (08-001607). All animal experiments performed were approved by the Mayo Clinic Institutional Animal Care and Use Committee (Mayo Clinic Institutional Animal Care and Use Committee protocols A6508, A48510).

### Reagents

A list of antibodies used in this study and their sources can be found in **Table S1**. Other reagents utilized: recombinant human TGF-α (Chemicon International), recombinant MMP-7 (Calbiochem), γ-secretase inhibitor (Tocris), soybean trypsin inhibitor (USB), Waymouth MB medium, Dexamethasone (Sigma Chemicals), Rat tail collagen (BD Biosciences), collagenase P (Roche), X-gal stock solution, Stain Base Solution and β-gal fixative (Millipore), adeno-null (control) virus, adeno-Cre virus and adeno-Cre-GFP virus (Vector BioLabs), aurothiomalate (Myochrysine; Taylor Pharmaceuticals).

#### *Mice*

*LSL-Kras^G12D^* (*LSL-Kras*) mice were obtained from the NCI Mouse Repository (MMHCC), *Rosa26* reporter (*R26R*) mice were obtained from Jackson Labs and *P48-Cre* mice were a gift from Dr. Pinku Mukherjee, University of North Carolina. *LSL-Kras* mice were crossed with *P48-Cre* mice to generate *P48-Cre;LSL-Kras* mice, as described by others. [2], [4] Floxed PKCι (*Prkci^f/f^*) mice (previously called floxed PKC-λ or *PKCλ^fl/fl^* mice) have been previously described. [18], [34], [40] In some experiments, previously described *LSL-Kras;Prkci^f/f^* mice were utilized. [16] Recombination of floxed alleles was characterized by PCR analysis of genomic DNA (see **Table S2** for PCR primer sequences).

#### Immunohistochemistry

Mouse tissues were processed for immunohistochemistry as described previously. [41] PKCι staining was visualized using Mouse-on-mouse HRP-Polymer kit (Biocare) and CK-19 was visualized using Rat-on-mouse HRP Polymer kit (Biocare). Images were captured and analyzed using Aperio and Spectrum software.

### Pancreatic acinar cell explant cultures

Mouse pancreatic acinar cells were isolated and cultured as described. [11], [14] Additional details can be found in **Supporting Materials and Methods S1.**

### Adenoviral infection and beta-galactosidase activity

Pancreatic acinar cells were infected with adeno-Cre, adeno-Cre-GFP or a control, adeno-null virus (50∶1 multiplicity of infection, MOI) overnight at 37°C, with gentle rocking every 15 minutes for the first hour. Thereafter, the cells were embedded in collagen matrix and grown for up to 7 days in explant culture. For detection of β-gal activity, collagen explants were washed, fixed and stained in X-gal overnight at 37°C. [11] Transduction efficiency calculation is described in **Supporting Materials and Methods S1.**

### Immunofluorescence

Pancreatic explant cultures were fixed and labeled with fluorescent antibodies as described. [11], [14] Fluorescent images were captured on a Zeiss LSM-510 Meta confocal microscope and bright field and GFP images were captured on an Olympus IX71/IX51 inverted microscope.

### Statistical analysis

Unless otherwise noted, two-way Analysis of Variance (ANOVA) was used to evaluate the statistical significance of the difference between groups, and a *P* value<.05 was considered statistically significant.

## Supporting Information

Figure S1 **PKCι expression and subcellular distribution in mPanINs.** PKCι expression detected by IHC (brown) in pancreata isolated from *P48-Cre*;*LSL*-*Kras* mice. Representative images of mPanINs and invasive adenocarcinoma are shown. Scale bar, 50 µm.
(TIF)

Figure S2 **Characterization of TGF-α-induced ADM.** **A**) Pancreatic acinar cells isolated from WT mice were embedded in collagen and treated with TGF-α. Scale bars, 100 µm. **B**) Co-immunofluorescence of the acinar cell marker amylase (red) and the ductal cell marker CK-19 (green) in day 1 and day 7 explant cultures. DAPI (blue) co-staining is shown. Scale bar, 25 µm.
(TIF)

Figure S3 **No effect of Cre-recombinase on TGF-α-induced ADM.** **A**) PCR analysis of genomic DNA detects recombined floxed *Prkci* allele in Ad-Cre-treated, but not control adenovirus-(Ad-null)-treated *Prkci^f/f^* mouse pancreatic acinar cells. See **Table S2** for PCR primer sequences. **B**) Representative bright field images of primary acinar cells from WT mice incubated with Ad-Cre and embedded in collagen ± TGF-α for 7 days. Scale bar, 200 µm. **C**) Pancreatic acinar cells were isolated from *R26R* mice, incubated with Ad-null or Ad-Cre and embedded in collagen ± TGF-α for 7 days. β-galactosidase staining indicates Ad-Cre-mediated recombination of the *ROSA26R* allele. Scale bar, 50 µm.
(TIF)

Figure S4 **Characterization of K-ras^G12D^-induced ADM.** **A**) PCR detection of recombined *LSL-Kras* allele in genomic DNA of Ad-Cre-treated *LSL-Kras* mouse pancreatic acinar cells. See **Table S2** for PCR primer sequences. **B**) Representative image of H&E stained, formalin-fixed, paraffin-embedded day 7 explant culture of Ad-Cre-treated *LSL-Kras* cells. Note the single layer of duct-like cells that surround the luminal structure is more easily distinguished in fixed and sectioned explant culture. **C**) Co-immunofluorescence of chymotrypsin (green) and carbonic anhydrase II (red) in Ad-Cre-treated LSL-Kras on day 1 and 7. DAPI (blue) staining is shown. **D**) Co-immunofluorescence of PKCι (red) and CK-19 (green) in Ad-Cre-treated *LSL-Kras* on day 7. DAPI (blue) staining is shown. Scale bar, 50 µm.
(TIF)

Figure S5 **Characterization of genetic and pharmacological inhibition of PKCι in primary acinar cells.** **A**) Co-immunofluorescence of PKCι (red) and CK-19 (green) in Ad-Cre-treated *LSL-Kras;Prkci^f/f^* cells in explant culture (day 7). DAPI (blue) staining is shown. Scale bar, 50 µm. **B**) Representative bright field and fluorescent images Adeno-Cre-GFP virus-treated *LSL-Kras* and *LSL-Kras;Prkci^f/f^* acinar cells in explant culture (day 6). GFP expression demonstrates high viral efficiency as well as cell viability. Scale bar, 200 µm. **C**) PCR detection of recombined *LSL-Kras* and floxed *Prkci* alleles in genomic DNA of Ad-Cre-treated pancreatic acinar cells, confirming Cre-recombinase activity. See **Table S2** for PCR primer sequences. **D**) Detection of PKCι (red) in Ad-Cre-treated *LSL-Kras* acinar cells in explant culture (day 7). Untreated (left panel) or+aurothiomalate (ATM; right panel). PKCι expression is elevated in ATM-treated cells, relative to non-K-ras^G12D^–expressing acinar cells (***panel A***), but cell-type-specific differences in PKCι subcellular distribution makes determination of relative PKCι expression in K-ras^G12D^–induced cells ± ATM (***panel D***), difficult. DAPI (blue) staining is shown. Scale bar, 25 µm. **E**) mRNA was isolated from day 1 and 6 explant cultures of Ad-Cre virus-treated *LSL-Kras* acinar cells +/− ATM and analyzed by qPCR for PKCι expression. Data is presented relative to 18 S abundance and presented relative to PKCι mRNA expression on day 1. Data presented is representative of two independent experiments.
(TIF)

Figure S6 **Characterization of the relationship between PKCι and MMP-7 in K-ras^G12D^-mediated ADM.** **A**) mRNA was isolated from day 1 and 6 explant cultures of Ad-Cre virus-treated *LSL-Kras* and *LSL-Kras;Prkci^f/f^* acinar cells and analyzed by qPCR for MMP-7 expression. Data is presented relative to 18 S abundance (×10^5^) and is representative of two independent experiments. **B**) Immunofluorescence of PKCι (red) in Ad-Cre-treated *LSL-Kras;Prkci^f/f^* cells plated with 200 ng/ml active recombinant MMP-7 (rMMP-7) in explant culture (day 6). DAPI (blue) staining is shown. Scale bar, 50 µm.
(TIF)

Table S1 **Summary of antibodies used.**
(DOC)

Table S2 **Summary of PCR primers.**
(DOC)

Materials and Methods S1 (DOC)

## References

[1] Klimstra DS, Longnecker DS (1994). K-ras mutations in pancreatic ductal proliferative lesions.. Am J Pathol.

[2] Hingorani SR, Petricoin EF, Maitra A, Rajapakse V, King C (2003). Preinvasive and invasive ductal pancreatic cancer and its early detection in the mouse.. Cancer Cell.

[3] Carriere C, Seeley ES, Goetze T, Longnecker DS, Korc M (2007). The Nestin progenitor lineage is the compartment of origin for pancreatic intraepithelial neoplasia.. Proc Natl Acad Sci U S A.

[4] Zhu L, Shi G, Schmidt CM, Hruban RH, Konieczny SF (2007). Acinar cells contribute to the molecular heterogeneity of pancreatic intraepithelial neoplasia.. Am J Pathol.

[5] Hruban RH, Maitra A, Goggins M (2008). Update on pancreatic intraepithelial neoplasia.. Int J Clin Exp Pathol.

[6] Slack JM (2007). Metaplasia and transdifferentiation: from pure biology to the clinic.. Nat Rev Mol Cell Biol.

[7] Wagner M, Luhrs H, Kloppel G, Adler G, Schmid RM (1998). Malignant transformation of duct-like cells originating from acini in transforming growth factor transgenic mice.. Gastroenterology.

[8] Meszoely IM, Means AL, Scoggins CR, Leach SD (2001). Developmental aspects of early pancreatic cancer.. Cancer J.

[9] Bockman DE, Merlino G (1992). Cytological changes in the pancreas of transgenic mice overexpressing transforming growth factor alpha.. Gastroenterology.

[10] Sandgren EP, Luetteke NC, Palmiter RD, Brinster RL, Lee DC (1990). Overexpression of TGF alpha in transgenic mice: induction of epithelial hyperplasia, pancreatic metaplasia, and carcinoma of the breast.. Cell.

[11] Means AL, Meszoely IM, Suzuki K, Miyamoto Y, Rustgi AK (2005). Pancreatic epithelial plasticity mediated by acinar cell transdifferentiation and generation of nestin-positive intermediates.. Development.

[12] Miyamoto Y, Maitra A, Ghosh B, Zechner U, Argani P (2003). Notch mediates TGF alpha-induced changes in epithelial differentiation during pancreatic tumorigenesis.. Cancer Cell.

[13] Crawford HC, Scoggins CR, Washington MK, Matrisian LM, Leach SD (2002). Matrix metalloproteinase-7 is expressed by pancreatic cancer precursors and regulates acinar-to-ductal metaplasia in exocrine pancreas.. J Clin Invest.

[14] Sawey ET, Johnson JA, Crawford HC (2007). Matrix metalloproteinase 7 controls pancreatic acinar cell transdifferentiation by activating the Notch signaling pathway.. Proc Natl Acad Sci U S A.

[15] Murray NR, Jamieson L, Yu W, Zhang J, Gokmen-Polar Y (2004). Protein kinase C{iota} is required for Ras transformation and colon carcinogenesis in vivo.. J Cell Biol.

[16] Regala RP, Davis RK, Kunz A, Khoor A, Leitges M (2009). Atypical protein kinase C{iota} is required for bronchioalveolar stem cell expansion and lung tumorigenesis.. Cancer Res.

[17] Scotti ML, Bamlet W, Smyrk TC, Fields AP, Murray NR (2010). Protein kinase C iota is required for pancreatic cancer cell transformed growth and tumorigenesis.. Cancer Res.

[18] Farese RV, Sajan MP, Yang H, Li P, Mastorides S (2007). Muscle-specific knockout of PKC-lambda impairs glucose transport and induces metabolic and diabetic syndromes.. J Clin Invest.

[19] Regala RP, Thompson EA, Fields AP (2008). Atypical protein kinase C iota expression and aurothiomalate sensitivity in human lung cancer cells.. Cancer Res.

[20] Stallings-Mann M, Jamieson L, Regala RP, Weems C, Murray NR (2006). A novel small-molecule inhibitor of protein kinase Ciota blocks transformed growth of non-small-cell lung cancer cells.. Cancer Res.

[21] Erdogan E, Lamark T, Stallings-Mann M, Lee J, Pellecchia M (2006). Aurothiomalate inhibits transformed growth by targeting the PB1 domain of protein kinase Ciota.. J Biol Chem.

[22] Heid I, Lubeseder-Martellato C, Sipos B, Mazur PK, Lesina M (2011). Rac1 Is Required for Development of Preneoplastic Lesions During Carcinogenesis in Mouse Pancreas.. Gastroenterology.

[23] De Strooper B, Annaert W, Cupers P, Saftig P, Craessaerts K (1999). A presenilin-1-dependent gamma-secretase-like protease mediates release of Notch intracellular domain.. Nature.

[24] Shearman MS, Beher D, Clarke EE, Lewis HD, Harrison T (2000). L-685,458, an aspartyl protease transition state mimic, is a potent inhibitor of amyloid beta-protein precursor gamma-secretase activity.. Biochemistry.

[25] Plentz R, Park JS, Rhim AD, Abravanel D, Hezel AF (2009). Inhibition of gamma-secretase activity inhibits tumor progression in a mouse model of pancreatic ductal adenocarcinoma.. Gastroenterology.

[26] De La OJ, Emerson LL, Goodman JL, Froebe SC, Illum BE (2008). Notch and Kras reprogram pancreatic acinar cells to ductal intraepithelial neoplasia.. Proc Natl Acad Sci U S A.

[27] Shi G, Zhu L, Sun Y, Bettencourt R, Damsz B (2009). Loss of the acinar-restricted transcription factor Mist1 accelerates Kras-induced pancreatic intraepithelial neoplasia.. Gastroenterology.

[28] Carriere C, Young AL, Gunn JR, Longnecker DS, Korc M (2009). Acute pancreatitis markedly accelerates pancreatic cancer progression in mice expressing oncogenic Kras.. Biochem Biophys Res Commun.

[29] Siveke JT, Einwachter H, Sipos B, Lubeseder-Martellato C, Kloppel G (2007). Concomitant pancreatic activation of Kras(G12D) and Tgfa results in cystic papillary neoplasms reminiscent of human IPMN.. Cancer Cell.

[30] Leach SD (2005). Epithelial differentiation in pancreatic development and neoplasia: new niches for nestin and Notch.. J Clin Gastroenterol.

[31] Habbe N, Shi G, Meguid RA, Fendrich V, Esni F (2008). Spontaneous induction of murine pancreatic intraepithelial neoplasia (mPanIN) by acinar cell targeting of oncogenic Kras in adult mice.. Proc Natl Acad Sci U S A.

[32] Mullendore ME, Koorstra JB, Li YM, Offerhaus GJ, Fan X (2009). Ligand-dependent Notch signaling is involved in tumor initiation and tumor maintenance in pancreatic cancer.. Clin Cancer Res.

[33] Hanlon L, Avila JL, Demarest RM, Troutman S, Allen M (2010). Notch1 functions as a tumor suppressor in a model of K-ras-induced pancreatic ductal adenocarcinoma.. Cancer Res.

[34] Murray NR, Weems J, Braun U, Leitges M, Fields AP (2009). Protein kinase C betaII and PKCiota/lambda: collaborating partners in colon cancer promotion and progression.. Cancer Res.

[35] Wilson CL, Heppner KJ, Labosky PA, Hogan BL, Matrisian LM (1997). Intestinal tumorigenesis is suppressed in mice lacking the metalloproteinase matrilysin.. Proc Natl Acad Sci U S A.

[36] van Es JH, van Gijn ME, Riccio O, van den Born M, Vooijs M (2005). Notch/gamma-secretase inhibition turns proliferative cells in intestinal crypts and adenomas into goblet cells.. Nature.

[37] Gomez del Pulgar T, Bandres E, Espina C, Valdes-Mora F, Perez-Palacios R (2007). Differential expression of Rac1 identifies its target genes and its contribution to progression of colorectal cancer.. Int J Biochem Cell Biol.

[38] Frederick LA, Matthews JA, Jamieson L, Justilien V, Thompson EA (2008). Matrix metalloproteinase-10 is a critical effector of protein kinase Ciota-Par6alpha-mediated lung cancer.. Oncogene.

[39] Regala RP, Weems C, Jamieson L, Copland JA, Thompson EA (2005). Atypical protein kinase Ciota plays a critical role in human lung cancer cell growth and tumorigenicity.. J Biol Chem.

[40] Calcagno SR, Li S, Shahid MW, Wallace MB, Leitges M (2011). Protein kinase C iota in the intestinal epithelium protects against dextran sodium sulfate-induced colitis.. Inflamm Bowel Dis.

[41] Calcagno SR, Li S, Colon M, Kreinest PA, Thompson EA (2008). Oncogenic K-ras promotes early carcinogenesis in the mouse proximal colon.. Int J Cancer.

