# Supplementary figures and images for: Protein Kinase C Iota Regulates Pancreatic Acinar-to-Ductal Metaplasia
Source: PLoS One. 2012 Feb 16;7(2):e30509. doi: 10.1371/journal.pone.0030509 (PMC3281025; doi:10.1371/journal.pone.0030509)

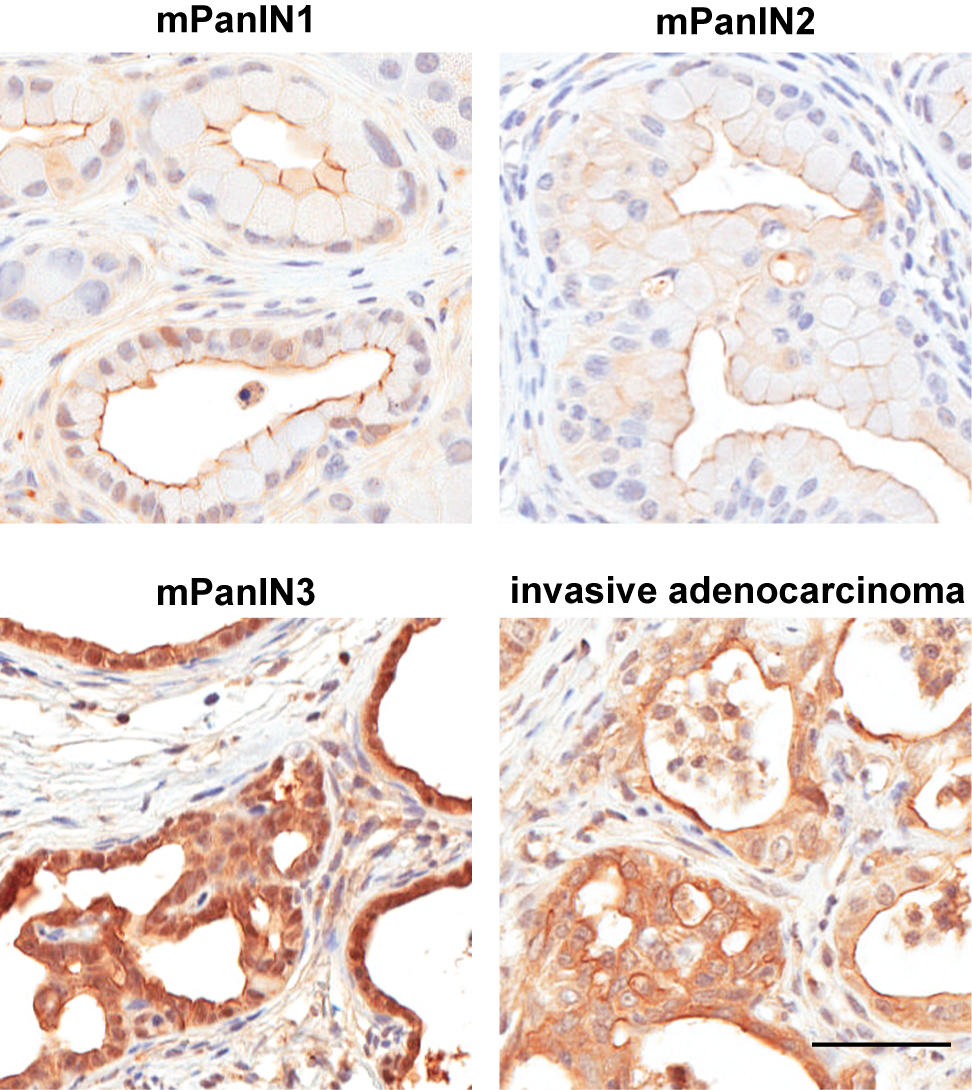

Supplement: Figure S1 — PKCι expression and subcellular distribution in mPanINs. PKCι expression detected by IHC (brown) in pancreata isolated from P48-Cre;LSL-Kras mice. Representative images of mPanINs and invasive adenocarcinoma are shown. Scale bar, 50 µm. (TIF) [file pone.0030509.s001.tif]

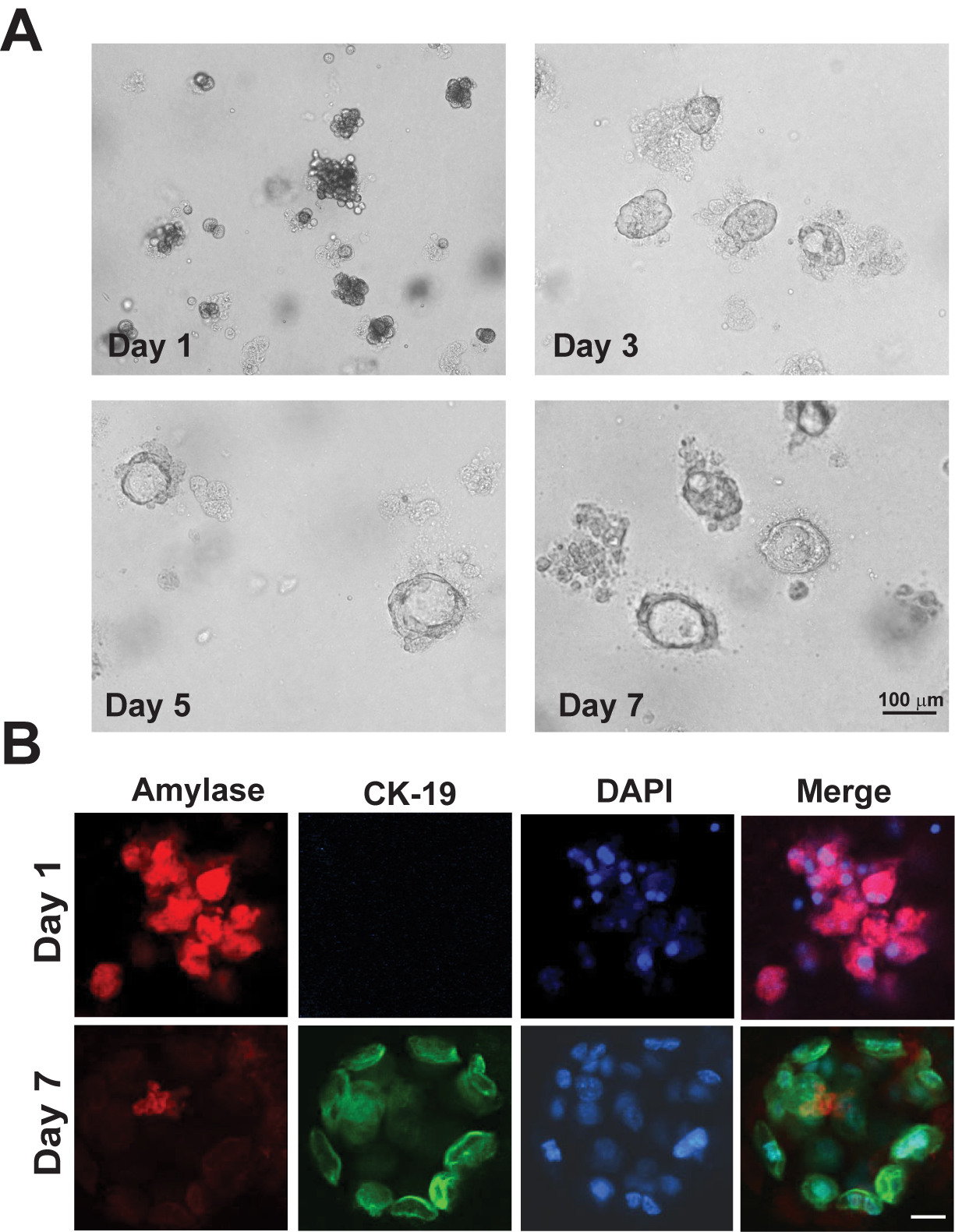

Supplement: Figure S2 — Characterization of TGF-α-induced ADM. A) Pancreatic acinar cells isolated from WT mice were embedded in collagen and treated with TGF-α. Scale bars, 100 µm. B) Co-immunofluorescence of the acinar cell marker amylase (red) and the ductal cell marker CK-19 (green) in day 1 and day 7 explant cultures. DAPI (blue) co-staining is shown. Scale bar, 25 µm. (TIF) [file pone.0030509.s002.tif]

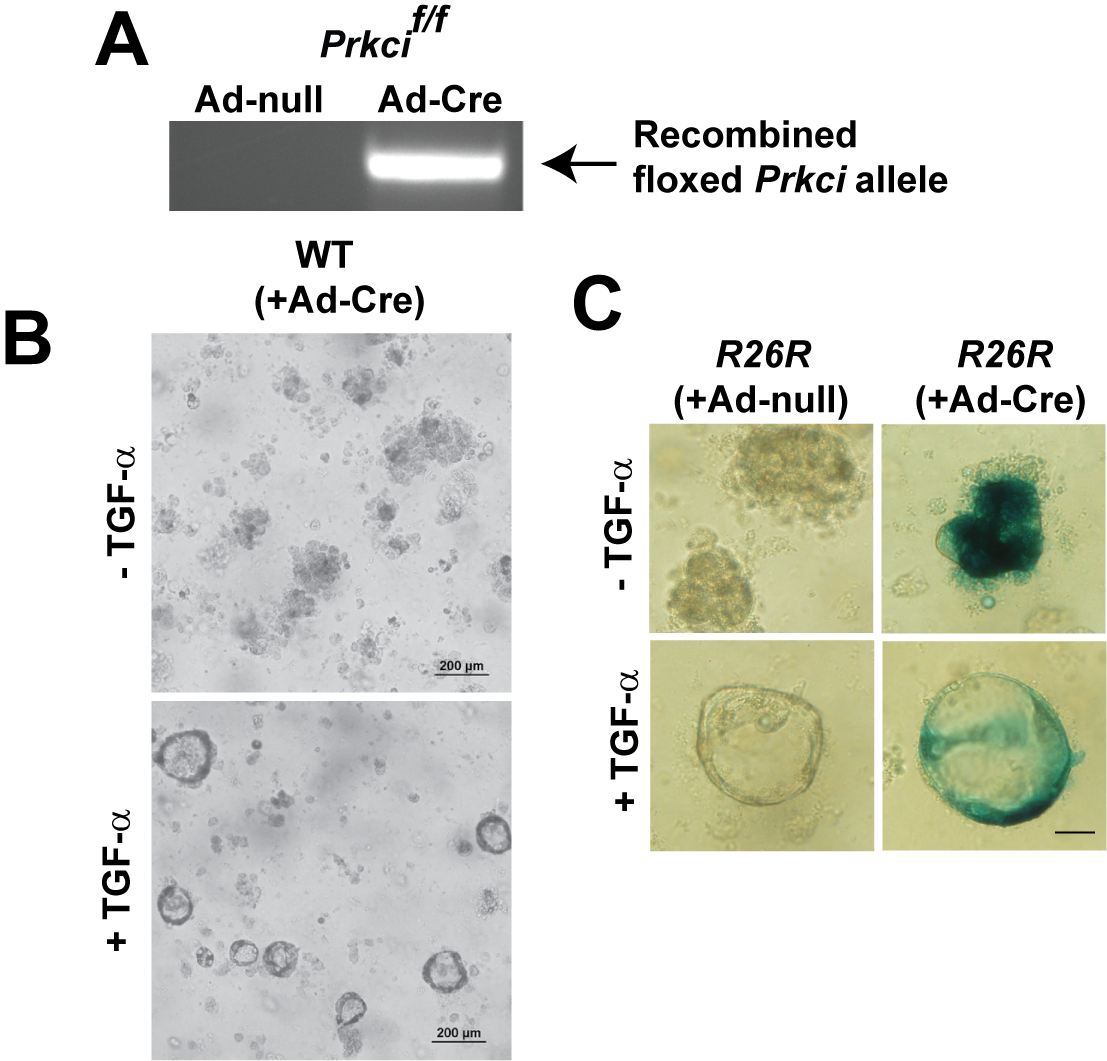

Supplement: Figure S3 — No effect of Cre-recombinase on TGF-α-induced ADM. A) PCR analysis of genomic DNA detects recombined floxed Prkci allele in Ad-Cre-treated, but not control adenovirus-(Ad-null)-treated Prkcif/f mouse pancreatic acinar cells. See Table S2 for PCR primer sequences. B) Representative bright field images of primary acinar cells from WT mice incubated with Ad-Cre and embedded in collagen ± TGF-α for 7 days. Scale bar, 200 µm. C) Pancreatic acinar cells were isolated from R26R mice, incubated with Ad-null or Ad-Cre and embedded in collagen ± TGF-α for 7 days. β-galactosidase staining indicates Ad-Cre-mediated recombination of the ROSA26R allele. Scale bar, 50 µm. (TIF) [file pone.0030509.s003.tif]

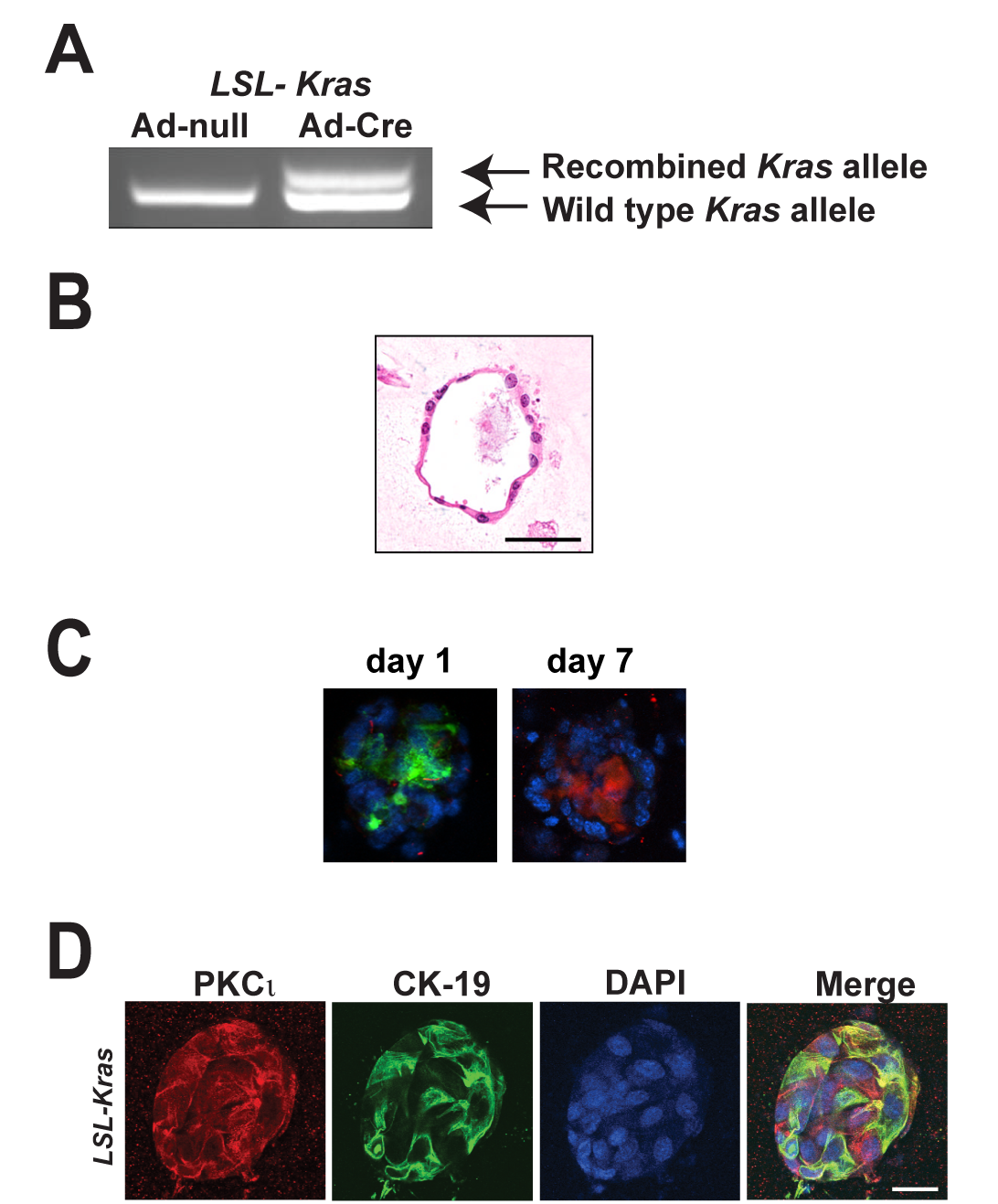

Supplement: Figure S4 — Characterization of K-rasG12D-induced ADM. A) PCR detection of recombined LSL-Kras allele in genomic DNA of Ad-Cre-treated LSL-Kras mouse pancreatic acinar cells. See Table S2 for PCR primer sequences. B) Representative image of H&E stained, formalin-fixed, paraffin-embedded day 7 explant culture of Ad-Cre-treated LSL-Kras cells. Note the single layer of duct-like cells that surround the luminal structure is more easily distinguished in fixed and sectioned explant culture. C) Co-immunofluorescence of chymotrypsin (green) and carbonic anhydrase II (red) in Ad-Cre-treated LSL-Kras on day 1 and 7. DAPI (blue) staining is shown. D) Co-immunofluorescence of PKCι (red) and CK-19 (green) in Ad-Cre-treated LSL-Kras on day 7. DAPI (blue) staining is shown. Scale bar, 50 µm. (TIF) [file pone.0030509.s004.tif]

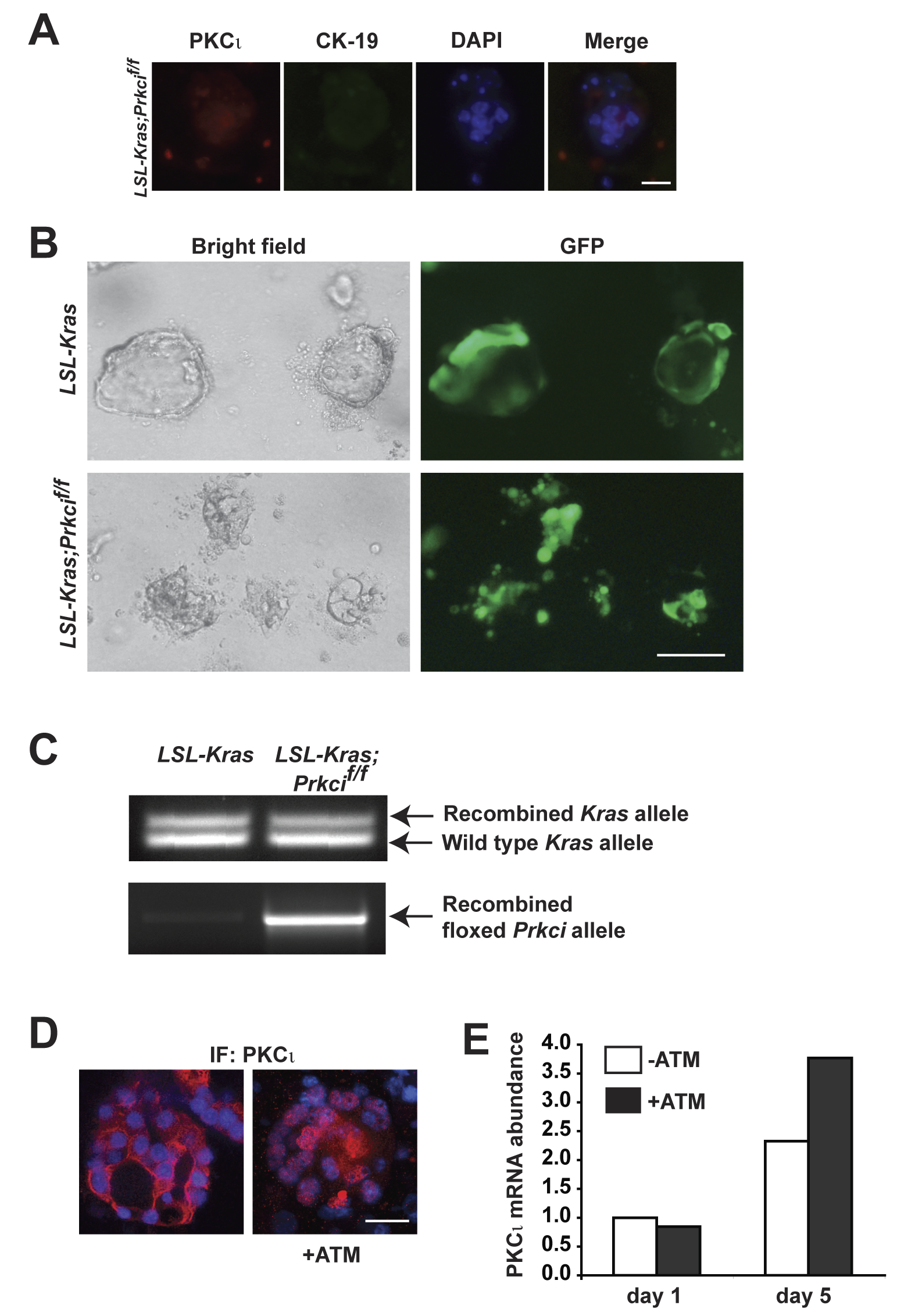

Supplement: Figure S5 — Characterization of genetic and pharmacological inhibition of PKCι in primary acinar cells. A) Co-immunofluorescence of PKCι (red) and CK-19 (green) in Ad-Cre-treated LSL-Kras;Prkcif/f cells in explant culture (day 7). DAPI (blue) staining is shown. Scale bar, 50 µm. B) Representative bright field and fluorescent images Adeno-Cre-GFP virus-treated LSL-Kras and LSL-Kras;Prkcif/f acinar cells in explant culture (day 6). GFP expression demonstrates high viral efficiency as well as cell viability. Scale bar, 200 µm. C) PCR detection of recombined LSL-Kras and floxed Prkci alleles in genomic DNA of Ad-Cre-treated pancreatic acinar cells, confirming Cre-recombinase activity. See Table S2 for PCR primer sequences. D) Detection of PKCι (red) in Ad-Cre-treated LSL-Kras acinar cells in explant culture (day 7). Untreated (left panel) or+aurothiomalate (ATM; right panel). PKCι expression is elevated in ATM-treated cells, relative to non-K-rasG12D–expressing acinar cells (panel A), but cell-type-specific differences in PKCι subcellular distribution makes determination of relative PKCι expression in K-rasG12D–induced cells ± ATM (panel D), difficult. DAPI (blue) staining is shown. Scale bar, 25 µm. E) mRNA was isolated from day 1 and 6 explant cultures of Ad-Cre virus-treated LSL-Kras acinar cells +/− ATM and analyzed by qPCR for PKCι expression. Data is presented relative to 18 S abundance and presented relative to PKCι mRNA expression on day 1. Data presented is representative of two independent experiments. (TIF) [file pone.0030509.s005.tif]

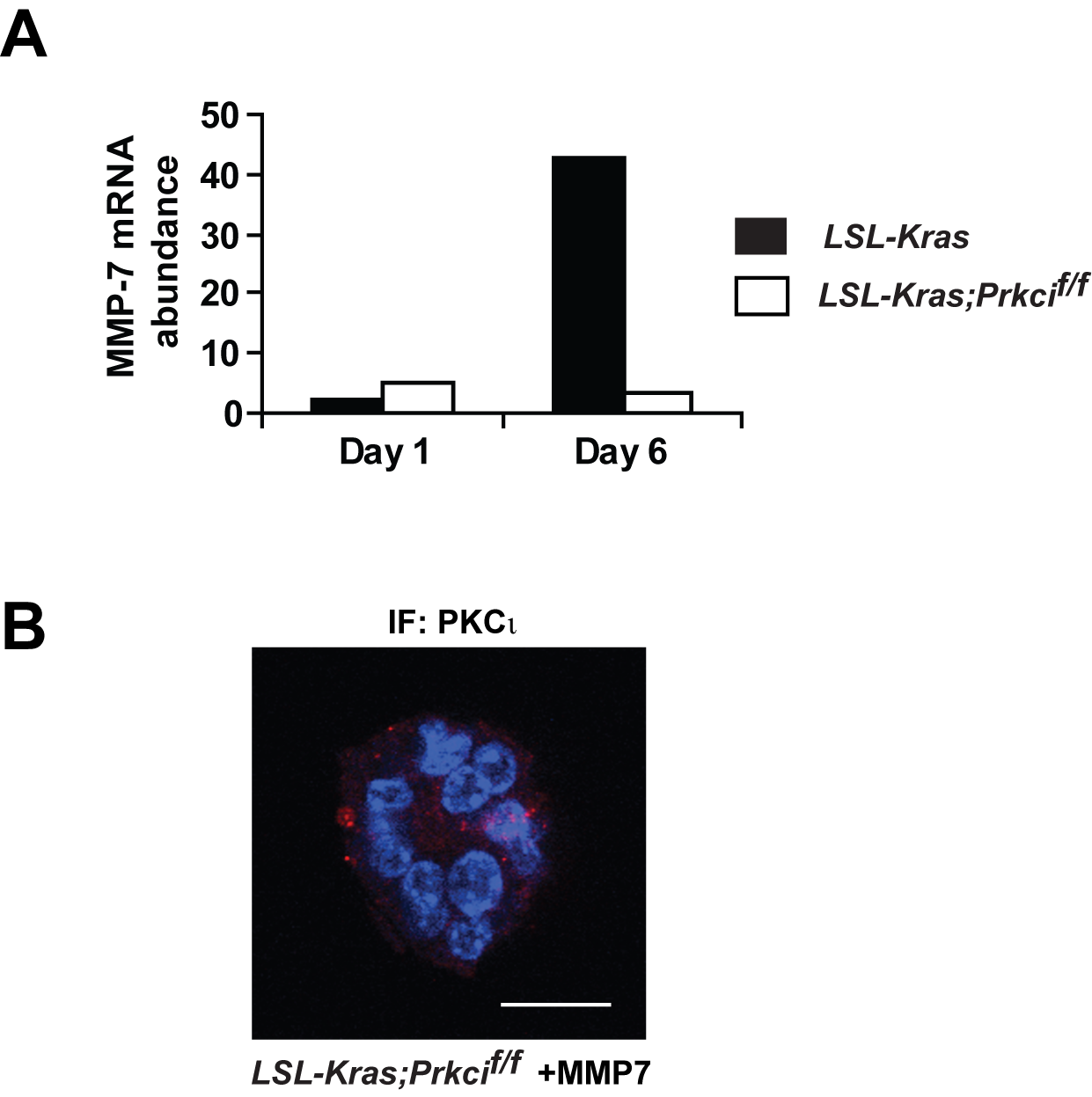

Supplement: Figure S6 — Characterization of the relationship between PKCι and MMP-7 in K-rasG12D-mediated ADM. A) mRNA was isolated from day 1 and 6 explant cultures of Ad-Cre virus-treated LSL-Kras and LSL-Kras;Prkcif/f acinar cells and analyzed by qPCR for MMP-7 expression. Data is presented relative to 18 S abundance (×105) and is representative of two independent experiments. B) Immunofluorescence of PKCι (red) in Ad-Cre-treated LSL-Kras;Prkcif/f cells plated with 200 ng/ml active recombinant MMP-7 (rMMP-7) in explant culture (day 6). DAPI (blue) staining is shown. Scale bar, 50 µm. (TIF) [file pone.0030509.s006.tif]
