# Supplementary material for: Protein Kinase C Iota Regulates Pancreatic Acinar-to-Ductal Metaplasia
Source: PLoS One. 2012 Feb 16;7(2):e30509. doi: 10.1371/journal.pone.0030509 (PMC3281025; doi:10.1371/journal.pone.0030509)
Supplement: Table S1 — Summary of antibodies used. (DOC) [file pone.0030509.s007.doc]

**Supplemental Table 1. Summary of antibodies used**

______________________________________________________________________

**Antibody Catalog # Source**

______________________________________________________________________

PKCι N-20, 727 Santa Cruz Biotechnology, Inc.

PKCι Clone 23 BD Biosciences

Matrix metalloproteinase-7 M8683 Sigma

Amylase A8273 Sigma

Cytokeratin-19 NCL-CK19 Novocastra Laboratories Ltd.

Cytokeratin-19 TROMAIII Dev. Studies Hybridoma Bank

Nestin 556309 BD Pharmingen

Cleaved Notch1 2421 Cell Signaling Technology

Hes-1 ab71559 Abcam

Chymotrypsin sc-80750 Santa Cruz

Carbonic Anhydrase II ab6621 Abcam

Goat anti-mouse IgG2b ab98703 Abcam

Goat anti-rabbit 594 A11012 Invitrogen

Goat anti-rabbit 488 A11008 Invitrogen

Goat anti-mouse 488 A11001 Invitrogen

______________________________________________________________
