# Supplementary material for: Protein Kinase C Iota Regulates Pancreatic Acinar-to-Ductal Metaplasia
Source: PLoS One. 2012 Feb 16;7(2):e30509. doi: 10.1371/journal.pone.0030509 (PMC3281025; doi:10.1371/journal.pone.0030509)
Supplement: Table S2 — Summary of PCR primers. (DOC) [file pone.0030509.s008.doc]

**Supplemental Table 2. Summary of PCR primers**

_______________________________________________________________________

**Position Primer Sequence**

________________________________________________________________________

*K-ras^G12D^* recombination

RECO1: 5’-GGGTAGGTGTTGGGATAGCTG-3’

RECO2: 5’-TCCGAATTCAGTGACTACAGATGTACAGAG-3’

*Prkci^f/f^* recombination

F2: 5’-AATTGTTCATGTTCAACACTGCT-3’

F4: 5’-ACTAAGCATTGCCTGGCATC-3’

________________________________________________________________________
