# Supplementary material for: Protein Kinase C Iota Regulates Pancreatic Acinar-to-Ductal Metaplasia
Source: PLoS One. 2012 Feb 16;7(2):e30509. doi: 10.1371/journal.pone.0030509 (PMC3281025; doi:10.1371/journal.pone.0030509)
Supplement: Materials and Methods S1 — (DOC) [file pone.0030509.s009.doc]

**SUPPORTING MATERIALS and METHODS**

**Calculation of Viral Transduction Efficiency.** Transduction efficiency of adeno-Cre virus was calculated by determining the percent of infected acinar cells (isolated from mice carrying the *R26R* allele) expressing β-gal activity. Transduction efficiency of adeno-Cre-GFP was calculated by determining the percent of infected acinar cells expressing GFP 24 hours after viral transduction. Each well was photographed at four random non-overlapping locations under bright field and fluorescent settings. The number of cells were counted in the bright field and fluorescent images and the percent of cells that were fluorescent (fluorescent cells/total bright field cells x100) was calculated to yield the viral transduction efficiency. The calculated transduction efficiency ranged from 85-95% and did not vary significantly from experiment to experiment. Transduction efficiency was not significantly altered by addition of growth factors or inhibitors.

**Acinar Cell Culture and Quantitation of Duct Formation.** Acinar cells were isolated from mouse pancreata (2-4 month old, male and female) and embedded in collagen matrix using a modification of previously described protocols.[11, 14] Briefly, the pancreas was minced, digested with collagenase, filtered successively through 500 μm and 105 μm mesh, separated by centrifugation through HBSS supplemented with 30% FBS at 1000 rpm x 2 min and plated in 24 well culture dishes. Growth factors and inhibitors (TGF-α, aurothiomalate, L-685,458, NSC23766, Erlotinib and rMMP-7) were added to the isolated acinar cells in the explant culture media. Explant culture media containing supplements, including rMMP-7 and ATM, was refreshed every other day. For quantitative analysis of duct formation, each well was photographed at four random non-overlapping locations at 4x magnification. The number of ducts in the four images were summed as the number of ducts per well. Three or more wells per experimental condition were counted and averaged.

**Isolation of mRNA and qPCR analysis.** Collagen disks were digested with 1 mg/ml collagenase in PBS at 37°C for 10-15 minutes with gentle shaking. The released cells were pelleted and lysed in RNAqueous solution (Ambion). Total RNA was isolated according to the manufacturer’s protocols. TaqMan® Gene Expression Assay primer and probe sets (Applied Biosystems) were used for real-time, quantitative PCR (qPCR) analysis of mouse MMP-7 (Mm00487724_m1), PKCι (Mm01293252_m1), TGF-α (Mm00446232_m1) and 18S (Hs99999901_s1). qPCR analysis was carried out using 2 ng cDNA on an Applied Biosystems 7900 thermal cycler. Data was evaluated using the SDS 2.3 software package. Gene expression was normalized to 18S. All data is expressed as 2-(*CT*(target)-*CT*(endogenous reference)).
